# Holistic Specifications for Robust Programs

**DOI:** 10.1007/978-3-030-45234-6_21

**Published:** 2020-03-13

**Authors:** Sophia Drossopoulou, James Noble, Julian Mackay, Susan Eisenbach

**Affiliations:** 8 University of Paderborn, Paderborn, Germany; 9 ICREA, Open University of Catalonia, Barcelona, Spain; 10 Imperial College London, London, United Kingdom; 11 Victoria University of Wellington, Wellington, New Zealand; 12 Microsoft Research Cambridge, Cambridge, United Kingdom

## Abstract

Functional specifications describe what program components *can* do: the *sufficient* conditions to invoke components’ operations. They allow us to reason about the use of components in a *closed world* setting, where components interact with known client code, and where the client code must establish the appropriate pre-conditions before calling into a component.

Sufficient conditions are not enough to reason about the use of components in an *open world* setting, where components interact with external code, possibly of unknown provenance, and where components may evolve over time. In this open world setting, we must also consider the *necessary* conditions, *i. e.* what are the conditions without which an effect will *not* happen.

In this paper we propose the ${\mathcal {C}}$hainmail specification language for writing *holistic* specifications that focus on necessary conditions (as well as sufficient conditions). We give a formal semantics for ${\mathcal {C}}$hainmail, and discuss several examples. The core of ${\mathcal {C}}$hainmail has been mechanised in the Coq proof assistant.

## 1 Introduction

Software guards our secrets, our money, our intellectual property, our reputation [47]. We entrust personal and corporate information to software which works in an *open* world, where it interacts with third party software of unknown provenance, possibly buggy and potentially malicious.

This means we need our software to be *robust*: to behave correctly even if used by erroneous or malicious third parties. We expect that our bank will only make payments from our account if instructed by us, or by somebody we have authorised, that space on a web page given to an advertiser will not be used to obtain access to our bank details [43], or that a concert hall will not book the same seat more than once.

While language mechanisms such as constants, invariants, object capabilities [40], and ownership [14] make it *possible* to write robust programs, they cannot *ensure* that programs are robust. Ensuring robustness is difficult because it means different things for different systems: perhaps that critical operations should only be invoked with the requisite authority; perhaps that sensitive personal information should not be leaked; or perhaps that a resource belonging to one user should not be consumed by another. To ensure robustness, we need ways to specify what robustness means for a particular

```
class Safe{
   field treasure
   field secret
   method take(scr){
      if (secret==scr) then
        {
        t=treasure
        treasure = null
        return t }   }
 }
```

```
class Safe{
   field treasure
   field secret
   method take(scr){
       ...as version 1...
   }
   method set(scr){
        secret=scr }
 }
```

**Figure 1.** Two Versions of the class Safe program, and ways to demonstrate that the particular program adheres to its specific robustness requirements.

Consider the code snippets from Fig. 1. Objects of class Safe hold a treasure and a secret, and only the holder of the secret can remove the treasure from the safe. We show the code in two versions; both have the same method take, and the second version has an additional method set. We assume a dynamically typed language (so that our results are applicable to both statically and dynamically typed settings)[4]; that fields are private in the sense of Java (*i.e.* only methods of that class may read or write these fields); and that addresses are unforgeable (so there is no way to guess a secret). A classical Hoare triple describing the behaviour of take would be:

(ClassicSpec) $\triangleq$
```
method take(scr)
PRE:   this:Safe
POST:  scr=this.secret_pre  ⟶ this.treasure=null
              ∧
       scr≠this.secret_pre  ⟶ ∀s:Safe. s.treasure=s.treasure_pre
```

(ClassicSpec) expresses that knowledge of the secret is *sufficient* to remove the treasure, and that take cannot remove the treasure unless the secret is provided. But it cannot preclude that Safe – or some other class, for that matter – contains more methods which might make it possible to remove the treasure without knowledge of the secret. This is the problem with the second version of Safe: it satisfies (ClassicSpec), but is not robust, as it is possible to overwrite the secret of the Safe and then use it to remove the treasure. To express robustness requirements, we introduce *holistic specifications*, and require that:

(HolisticSpec) $\triangleq$

$$\forall \mathtt{s}.[\ \mathtt{s} : \mathtt{Safe} \land \mathtt{s.treasure} \neq \mathtt{null} \land \mathsf{will}\langle\, \mathtt{s.treasure} = \mathtt{null}\,\rangle \longrightarrow\ \exists \mathtt{o}.[\,\mathsf{external}\langle\, \mathtt{o}\,\rangle \land \langle\, \mathtt{o}\ \mathsf{access}\ \mathtt{s.secret}\,\rangle\,]\ ]$$

(HolisticSpec) mandates that for any safe s whose treasure is not null, if some time in the future its treasure were to become null, then at least one external object (*i.e.* an object whose class is not Safe) in the current configuration has direct access

[4] We do not depend on the additional safety static typing provides, so we assume only a dynamically typed language.

to `s`'s secret. This external object need not have caused the change in `s.treasure` but it would have (transitively) passed access to the secret which ultimately did cause that change. Both classes in Fig. 1 satisfy (ClassicSpec), but the second version does not satisfy (HolisticSpec).

In this paper we propose *Chainmail*, a specification language to express holistic specifications. The design of *Chainmail* was guided by the study of a sequence of examples from the object-capability literature and the smart contracts world: the membrane [17], the DOM [20,59], the Mint/Purse [40], the Escrow [18], the DAO [12,15] and ERC20 [61]. As we worked through the examples, we found a small set of language constructs that let us write holistic specifications across a range of different contexts. *Chainmail* extends traditional program specification languages [31,37] with features which talk about:

**Permission:** Which objects may have access to which other objects; this is central since access to an object grants access to the functions it provides.

**Control:** Which objects called functions on other objects; this is useful in identifying the causes of certain effects - eg funds can only be reduced if the owner called a payment function.

**Time:** What holds some time in the past, the future, and what changes with time,

**Space:** Which parts of the heap are considered when establishing some property, or when performing program execution; a concept related to, but different from, memory footprints and separation logics,

**Viewpoint:** Which objects and which configurations are internal to our component, and which are external to it; a concept related to the open world setting.

While many individual features of *Chainmail* can be found in other work, their power and novelty for specifying open systems lies in their careful combination. The contributions of this paper are:

- the design of the holistic specification language *Chainmail*,
- the semantics of *Chainmail*,
- a Coq mechanisation of the core of *Chainmail*.

The rest of the paper is organised as follows: Section 2 gives an example from the literature which we will use to elucidate key points of *Chainmail*. 3 presents the *Chainmail* specification language. Section 4 introduces the formal model underlying *Chainmail*, and then section 5 defines the semantics of *Chainmail*'s assertions. Section 6 discusses our design, 7 considers related work, and section 8 concludes. We relegate key points of exemplar problems and various details to appendices which are available at [1].

## 2 Motivating Example: The Bank

As a motivating example, we consider a simplified banking application taken from the object capabilities literature [41]: `Accounts` belong to `Banks` and hold money (`balances`); with access to two `Accounts` of the same `Bank` one can transfer any amount of money from one to the other. This example has the advantage that it requires several objects and classes.

We will not show the code here (see appendix C), but suffice it to say that class `Account` has methods `deposit(src, amt)` and `makeAccount(amt)` (*i.e.* a method called `deposit` with two arguments, and a method called `makeAccount` with one argument). Similarly, `Bank` has method `newAccount(amt)`. Moreover, `deposit` requires that the receiver and first argument (`this` and `src`) are `Accounts` and belong to the same bank, that the second argument (`amt`) is a number, and that `src`'s balance is at least `amt`. If this condition holds, then `amt` gets transferred from `src` to the receiver. The function `makeNewAccount` returns a fresh `Account` with the same bank, and transfers `amt` from the receiver `Account` to the new `Account`. Finally, the function `newAccount` when run by a `Bank` creates a new `Account` with corresponding amount of money in it.[5] It is not difficult to give formal specifications of these methods in terms of pre- and post-conditions.

However, what if the bank provided a `steal` method that emptied out every account in the bank into a thief's account? The critical problem is that a bank implementation including a `steal` method could meet the functional specifications of `deposit`, `makeAccount`, and `newAccount`, and still allow the clients' money to be stolen.

One obvious solution would be to adopt a closed-world interpretation of specifications: we interpret functional specifications as *exact* in the sense that only implementations that meet the functional specification exactly, *with no extra methods or behaviour*, are considered as suitable implementations of the functional specification. The problem is that this solution is far too strong: it would for example rule out a bank that during software maintenance was given a new method `count` that simply counted the number of deposits that had taken place, or a method `notify` to enable the bank to occasionally send notifications to its customers.

What we need is some way to permit bank implementations that send notifications to customers, but to forbid implementations of `steal`. The key here is to capture the (implicit) assumptions underlying the design of the banking application. We provide additional specifications that capture those assumptions. The following three informal requirements prevent methods like `steal`:

1. An account's balance can be changed only if a client calls the `deposit` method with the account as the receiver or as an argument.
2. An account's balance can be changed only if a client has access to that particular account.
3. The `Bank`/`Account` component does not leak access to existing accounts or banks.

Compared with the functional specification we have seen so far, these requirements capture *necessary* rather than *sufficient* conditions: Calling the `deposit` method to gain access to an account is necessary for any change to that account taking place. The function `steal` is inconsistent with requirement (1), as it reduces the balance of an `Account` without calling the function `deposit`. However, requirement (1) is not enough to protect our money. We need (2) to avoid an `Account`'s balance getting

[5] Note that our very limited bank specification doesn't even have the concept of an account owner.

modified without access to the particular Account, and (3) to ensure that such accesses are not leaked.

We can express these requirements through *Chainmail* assertions. Rather than specifying the behaviour of particular methods when they are called, we write assertions that range across the entire behaviour of the Bank/Account module:

$$(1) \triangleq \forall \texttt{a}.[\ \texttt{a}:\texttt{Account} \wedge \textsf{changes}\langle\, \texttt{a.balance}\,\rangle \ \longrightarrow \\ \exists \texttt{o}.[\ \langle\, \texttt{o}\ \textsf{calls}\ \texttt{a.deposit(\_,\_)}\ \rangle \vee \langle\, \texttt{o}\ \textsf{calls}\ \texttt{\_.deposit(a,\_)}\ \rangle\ ]\ \ ]$$

$$(2) \triangleq \forall \texttt{a}.\forall \texttt{S}:\texttt{Set}.[\ \texttt{a}:\texttt{Account} \ \wedge\ \langle \textsf{will}\langle\ \textsf{changes}\langle\, \texttt{a.balance}\,\rangle\rangle\ \textsf{in}\ \texttt{S}\ \rangle \\ \longrightarrow\ \ \exists \texttt{o}.\ [\texttt{o} \in \texttt{S}\ \wedge\ \textsf{external}\langle\, \texttt{o}\,\rangle\ \wedge\ \langle\, \texttt{o}\ \textsf{access}\ \texttt{a}\,\rangle]\ \ ]$$

$$(3) \triangleq \forall \texttt{a}.\forall \texttt{S}:\texttt{Set}.[\ \texttt{a}:\texttt{Account} \wedge \\ \langle \textsf{will}\langle\ \exists \texttt{o}.[\ \textsf{external}\langle\, \texttt{o}\,\rangle\ \wedge\ \langle\, \texttt{o}\ \textsf{access}\ \texttt{a}\,\rangle]\,\rangle\ \textsf{in}\ \texttt{S}\,\rangle \\ \longrightarrow\ \ \exists \texttt{o}'.\ [\texttt{o}' \in \texttt{S}\ \wedge\ \textsf{external}\langle\, \texttt{o}'\,\rangle\ \wedge\ \langle\, \texttt{o}'\ \textsf{access}\ \texttt{a}\,\rangle\,]\ \ ]$$

In the above and throughout the paper, we use an underscore (_) to indicate an existentially bound variable whose value is of no interest.

Assertion (1) says that if an account's balance changes (changes⟨ a.balance ⟩), then there must be some client object o that called the deposit method with a as a receiver or as an argument (⟨ o calls _.deposit( _) ⟩).

Assertion (2) similarly constrains any possible change to an account's balance. If at some future point the balance changes (will⟨ changes⟨ ... ⟩ ⟩), and if this future change is observed with the state restricted to the objects from S (*i.e.* ⟨ ... in S ⟩), then at least one of these objects ($\texttt{o} \in \texttt{S}$) is external to the Bank/Account system (external⟨ o ⟩) and has direct access to that account object (⟨ o access a ⟩). Notice that while the change in the balance happens some time in the future, the external object o has access to a in the *current* state. Notice also that the object which makes the call to deposit described in (1), and the object which has access to a in the current state described in (2), need not be the same: it may well be that the latter passes a reference to a to the former (indirectly), which then makes the call to deposit.

It remains to think about how access to an Account may be obtained. This is the remit of assertion (3), which says that if at some time in the future of the state restricted to S, some object o which is external has access to some account a, and if a exists in the current state, then in the current state some object from S has access to a. Where o and o′ may, but need not, be the same object. And where o′ has to exist and have access to a in the *current* state, but o need not exist in the current state – it may be allocated later. Assertion (3) thus gives essential protection when dealing with foreign, untrusted code. When an Account is given out to untrusted third parties, assertion (3) guarantees that this Account cannot be used to obtain access to further Accounts.

A holistic specification for the bank account, then, would be a sufficient functional specification plus the necessary specifications (1)-(3) from above. This holistic specification permits an implementation of the bank that also provides count and notify methods, even though the specification does not mention either method. Critically, though, the holistic *Chainmail* specification does not permit an implementation that includes a steal method.

## 3 *Chainmail* Overview

In this Section we give a brief and informal overview of some of the most salient features of *Chainmail*– a full exposition appears in Section 5.

*Example Configurations* We will illustrate these features using the `Bank/Account` example from the previous section. We use the runtime configurations $\sigma_1$ and $\sigma_2$ shown in the left and right diagrams in Figure 2. In both diagrams the rounded boxes depict objects: green for those from the `Bank/Account` component, and grey for the "external", "client" objects. The transparent green rectangle shows which objects are contained by the `Bank/Account` component. The object at `1` is a `Bank`, those at `2`, `3` and `4` are `Accounts`, and those at `91`, `92`, `93` and `94` are "client" objects which belong to classes different from those from the `Bank/Account` module.

Each configuration represents one alternative implementation of the Bank object. Configuration $\sigma_1$ may arise from execution using a module $M_{BA1}$, where `Account` objects have a field `myBank` pointing to their `Bank`, and an integer field `balance` – the code can be found in appendix C Fig. 3. Configuration $\sigma_2$ may arise from execution using a module $M_{BA2}$, where `Accounts` have a `myBank` field, `Bank` objects have a `ledger` implemented though a sequence of `Nodes`, each of which has a field pointing to an `Account`, a field `balance`, and a field `next` – the code can be found in appendix C Figs. 6 and 4.

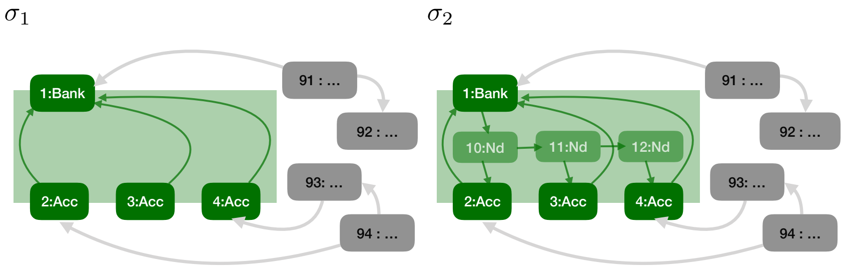

**Figure 2.** Two runtime configurations for the `Bank/Account` example.

For the rest, assume variable identifiers $\mathtt{b}_1$, and $\mathtt{a}_2$–$\mathtt{a}_4$, and $\mathtt{u}_{91}$–$\mathtt{u}_{94}$ denoting objects `1`, `2–4`, and `91–94` respectively for both $\sigma_1$ and $\sigma_2$. That is, for $i$=1 or $i$=2, $\sigma_i(\mathtt{b}_1)$=`1`, $\sigma_i(\mathtt{a}_2)$=`2`, $\sigma_i(\mathtt{a}_3)$=`3`, $\sigma_i(\mathtt{a}_4)$=`4`, $\sigma_i(\mathtt{u}_{91})$=`91`, $\sigma_i(\mathtt{u}_{92})$=`92`, $\sigma_i(\mathtt{u}_{93})$=`93`, and $\sigma_i(\mathtt{u}_{94})$=`94`.

*Classical Assertions* talk about the contents of the local variables (*i.e.* the topmost stack frame), and the fields of the various objects (*i.e.* the heap). For example, the assertion $\mathtt{a}_2$`.myBank=`$\mathtt{a}_3$`.myBank`, says that $\mathtt{a}_2$ and $\mathtt{a}_3$ have the same bank. In fact, this assertion is satisfied in both $\sigma_1$ and $\sigma_2$, written formally as

$$
\begin{aligned}
..., \sigma_1 &\models \mathtt{a}_2.\mathtt{myBank} = \mathtt{a}_3.\mathtt{myBank}\\
..., \sigma_2 &\models \mathtt{a}_2.\mathtt{myBank} = \mathtt{a}_3.\mathtt{myBank}.
\end{aligned}
$$

The term $\texttt{x}$:$\texttt{ClassId}$ says that $\texttt{x}$ is an object of class $\texttt{ClassId}$. For example

$...,\sigma_1 \models \texttt{a}_2.\texttt{myBank} : \texttt{Bank}.$

We support ghost fields [11,31], *e.g.* $\texttt{a}_1.\texttt{balance}$ is a physical field in $\sigma_1$ and a ghost field in $\sigma_2$ since in MBA2 an $\texttt{Account}$ does not store its $\texttt{balance}$ (as can be seen in appendix C Fig. 6). We also support the usual logical connectives, and so, we can express assertions such as

$\forall \texttt{a}.[\ \texttt{a} : \texttt{Account} \longrightarrow \texttt{a.myBank} : \texttt{Bank} \wedge \texttt{a.balance} \geq 0\ ].$

*Permission: Access* Our first holistic assertion, $\langle\, \texttt{x}\ \textsf{access}\ \texttt{y}\,\rangle$, asserts that object $\texttt{x}$ has a direct reference to another object $\texttt{y}$: either one of $\texttt{x}$'s fields contains a reference to $\texttt{y}$, or the receiver of the currently executing method is $\texttt{x}$, and $\texttt{y}$ is one of the arguments or a local variable. For example:

$...,\sigma_1 \models \langle\, \texttt{a}_2\ \textsf{access}\ \texttt{b}_1\,\rangle$

If $\sigma_1$ were executing the method body corresponding to the call $\texttt{a}_2.\texttt{deposit}(\texttt{a}_3,360)$, then we would have

$...,\sigma_1 \models \langle\, \texttt{a}_2\ \textsf{access}\ \texttt{a}_3\,\rangle,$

That is, during execution of $\texttt{deposit}$, the object at $\texttt{a}_2$ has access to the object at $\texttt{a}_3$, and could, if the method body chose to, call a method on $\texttt{a}_3$ , or store a reference to $\texttt{a}_3$ in its own fields. Access is not symmetric, nor transitive:

$...,\sigma_1 \not\models \langle\, \texttt{a}_3\ \textsf{access}\ \texttt{a}_2\,\rangle,$

$...,\sigma_2 \models \langle\, \texttt{a}_2\ \textsf{access}^*\ \texttt{a}_3\,\rangle, \qquad ...,\sigma_2 \not\models \langle\, \texttt{a}_2\ \textsf{access}\ \texttt{a}_3\,\rangle.$

*Control: Calls* The assertion $\langle\, \texttt{x}\ \textsf{calls}\ \texttt{m.y(}\ \texttt{zs)}\ \rangle$ holds in configurations where a method on object $\texttt{x}$ makes a method call $\texttt{y.m(zs)}$ — that is it calls method $\texttt{m}$ with object $\texttt{y}$ as the receiver, and with arguments $\texttt{zs}$. For example,

$...,\sigma_3 \models \langle\, \texttt{x}\ \textsf{calls}\ \texttt{a}_2.\texttt{deposit(}\ \texttt{a}_3, 360)\ \rangle.$

means that the receiver in $\sigma_3$ is $\texttt{x}$, and that $\texttt{a}_2.\texttt{deposit}(\texttt{a}_3, 360)$ is the next statement to be executed.

*Space: In* The space assertion $\langle\, A\ \textsf{in}\ \texttt{S}\,\rangle$ establishes validity of $A$ in a configuration restricted to the objects from the set $\texttt{S}$. For example, if object $\texttt{94}$ is included in $\texttt{S}_1$ but not in $\texttt{S}_2$, then we have

$...,\sigma_1 \models \langle\, (\exists \texttt{o}.\ \langle\, \texttt{o}\ \textsf{access}\ \texttt{a}_4\,\rangle)\ \textsf{in}\ \texttt{S}_1\,\rangle$

$...,\sigma_1 \not\models \langle\, (\exists \texttt{o}.\ \langle\, \texttt{o}\ \textsf{access}\ \texttt{a}_4\,\rangle)\ \textsf{in}\ \texttt{S}_2\,\rangle.$

The set $\texttt{S}$ in the assertion $\langle\, A\ \textsf{in}\ \texttt{S}\,\rangle$ is therefore *not* the footprint of $A$; it is more like the *fuel* [2] given to establish that assertion. Note that $...,\sigma \models \langle\, A\ \textsf{in}\ \texttt{S}\,\rangle$ does not imply $...,\sigma \models A$ nor does it imply $...,\sigma \models \langle\, A\ \textsf{in}\ \texttt{S} \cup \texttt{S}'\,\rangle$. The other direction of the implication does not hold either.

*Time: Next, Will, Prev, Was* We support several operators from temporal logic: ($\textsf{next}\langle\, A\,\rangle$, $\textsf{will}\langle\, A\,\rangle$, $\textsf{prev}\langle\, A\,\rangle$, and $\textsf{was}\langle\, A\,\rangle$) to talk about the future or the past in one or more steps. The assertion $\textsf{will}\langle\, A\,\rangle$ expresses that $A$ will hold in one or more steps. For example, taking $\sigma_4$ to be similar to $\sigma_2$, the next statement to be executed to be $\texttt{a}_2.\texttt{deposit}(\texttt{a}_3, 360)$, and $\texttt{M}_{BA2}\,⨾\,...,\sigma_4 \models \texttt{a}_2.\texttt{balance} = 60$, and that $\texttt{M}_{BA2}\,⨾\,...,\sigma_4 \models \texttt{a}_4.\texttt{balance} \geq 360$, then

$\texttt{M}_{BA2}\,⨾\,...,\sigma_4 \models \textsf{will}\langle\, \texttt{a}_2.\texttt{balance} = 420\,\rangle.$

The *internal* module, $\texttt{M}_{BA2}$ is needed for looking up the method body of $\texttt{deposit}$.

*Viewpoint: – External* The assertion $\mathsf{external}\langle\, \mathtt{x}\,\rangle$ expresses that the object at $\mathtt{x}$ does not belong to the module under consideration. For example,

$$\mathtt{M}_{AB2}\,⨾\,...,\sigma_2 \models \mathsf{external}\langle\, \mathtt{u}_{92}\,\rangle, \qquad \mathtt{M}_{AB2}\,⨾\,...,\sigma_2 \not\models \mathsf{external}\langle\, \mathtt{a}_2\,\rangle,$$
$$\mathtt{M}_{AB2}\,⨾\,...,\sigma_2 \not\models \mathsf{external}\langle\, \mathtt{b}_1\mathtt{.ledger}\,\rangle$$

The *internal* module, $\mathtt{M}_{BA2}$, is needed to judge which objects are internal or external.

*Change and Authority:* We have used $\mathsf{changes}\langle\, ...\,\rangle$ in our $\mathcal{C}hainmail$ assertions in section 2, as in $\mathsf{changes}\langle\, \mathtt{a.balance}\,\rangle$. Assertions that talk about change, or give conditions for change to happen are fundamental for security; the ability to cause change is called *authority* in [40]. We can encode change using the other features of $\mathcal{C}hainmail$, namely, for any expression $\mathtt{e}$:

$$\mathsf{changes}\langle\, \mathtt{e}\,\rangle \;\equiv\; \exists v.[\, \mathtt{e} = v \wedge \mathsf{next}\langle\, \neg(\mathtt{e} = v)\,\rangle\,].$$

and similarly for assertions.

*Putting these together* We now look at some composite assertions which use several features from above. For example, the assertion below says that if the statement to be executed is $\mathtt{a}_2\mathtt{.deposit(a}_3\mathtt{,60)}$, then the balance of $\mathtt{a}_2$ will eventually change:

$$\mathtt{M}_{BA2}⨾...,\sigma_2 \models \langle\, ..\,\mathsf{calls}\, \mathtt{a}_2\mathtt{.deposit(}\, \mathtt{a}_3\mathtt{,60)}\,\rangle \longrightarrow \mathsf{will}\langle\, \mathsf{changes}\langle\, \mathtt{a}_2\mathtt{.balance}\,\rangle\,\rangle.$$

Now look deeper into space assertions, $\langle\, A\, \mathsf{in}\, \mathtt{S}\,\rangle$, which allow us to characterise the set of objects which have authority over certain effects (here $A$). In particular, the assertion $\langle\, \mathsf{will}\langle\, A\,\rangle\, \mathsf{in}\, \mathtt{S}\,\rangle$ requires two things: i) that $A$ will hold in the future, and ii) that all the objects which cause the effect which will make $A$ valid are included in $\mathtt{S}$. Knowing who has, and who has not, authority over properties or data is a fundamental concern of robustness [40]. Notice that the authority is a set, rather than a single object: quite often it takes *several objects in concert* to achieve an effect.

Consider assertions (2) and (3) from the previous section. They both have the form "$\mathsf{will}\langle\, \langle\, A\, \mathsf{in}\, \mathtt{S}\,\rangle\,\rangle \longrightarrow P(\mathtt{S})$", where $P$ is some property over a set. These assertions say that if ever in the future $A$ becomes valid, and if the objects involved in making $A$ valid are included in $\mathtt{S}$, then $\mathtt{S}$ must satisfy $P$. Such assertions can be used to restrict whether $A$ will become valid. If we have some execution which only involves objects which do not satisfy $P$, then we know that the execution will not ever make $A$ valid.

*In summary,* in addition to classical logical connectors and classical assertions over the contents of the heap and the stack, our holistic assertions draw from some concepts from object capabilities ($\langle\, \_\, \mathsf{access}\, \_\,\rangle$ for permission; $\langle\, \_\, \mathsf{calls}\, \_.\_(\,\_)\,\rangle$ and $\mathsf{changes}\langle\, \_\,\rangle$ for authority) as well as temporal logic ($\mathsf{will}\langle\, A\,\rangle$, $\mathsf{was}\langle\, A\,\rangle$ and friends), and the relation of our spatial connective ($\langle\, A\, \mathsf{in}\, S\,\rangle$) with ownership and effect systems [60,14,13].

The next two sections discuss the semantics of $\mathcal{C}hainmail$. Section 4 contains an overview of the formal model and section 5 focuses on the most important part of $\mathcal{C}hainmail$: assertions.

## 4 Overview of the Formal foundations

We now give an overview of the formal model for $\mathcal{C}hainmail$. In section 4.1 we introduce the shape of the judgments used to give semantics to $\mathcal{C}hainmail$, while in section

4.2 we describe the most salient aspects of an underlying programming language used in $\mathcal{C}hainmail$.

### 4.1 $\mathcal{C}hainmail$ judgments

Having outlined the ingredients of our holistic specification language, the next question to ask is: When does a module M satisfy a holistic assertion $A$? More formally: when does $\texttt{M} \models A$ hold?

Our answer has to reflect the fact that we are dealing with an *open world*, where M, our module, may be linked with *arbitrary untrusted code*. To model the open world, we consider pairs of modules, $\texttt{M} \circ\!\!\!\!\!, \texttt{M}'$, where M is the module whose code is supposed to satisfy the assertion, and $\texttt{M}'$ is another module which exercises the functionality of M. We call our module M the *internal* module, and $\texttt{M}'$ the *external* module, which represents potential attackers or adversaries.

We can now answer the question: $\texttt{M} \models A$ holds if for all further, *potentially adversarial*, modules $\texttt{M}'$ and in all runtime configurations $\sigma$ which may be observed as arising from the execution of the code of M combined with that of $\texttt{M}'$, the assertion $A$ is satisfied. More formally, we define:

$$\texttt{M} \models A \qquad \text{if} \qquad \forall \texttt{M}'.\forall\sigma \in \mathcal{A}rising(\texttt{M} \,;\, \texttt{M}').[\; \texttt{M} \,;\, \texttt{M}', \sigma \models A \;].$$

Module $\texttt{M}'$ represents all possible clients of M. As it is arbitrarily chosen, it reflects the open world nature of our specifications.

The judgement $\texttt{M} \,;\, \texttt{M}', \sigma \models A$ means that assertion $A$ is satisfied by $\texttt{M} \,;\, \texttt{M}'$ and $\sigma$. As in traditional specification languages [31,37], satisfaction is judged in the context of a runtime configuration $\sigma$; but in addition, it is judged in the context of the internal and external modules. These are used to find abstract functions defining ghost fields as well as method bodies needed when judging validity of temporal assertions such as $\mathsf{will}\langle\_\rangle$.

We distinguish between internal and external modules. This has two uses: First, $\mathcal{C}hainmail$ includes the "$\mathsf{external}\langle\, o \,\rangle$" assertion to require that an object belongs to the external module, as in the Bank Account's assertion (2) and (3) in section 2. Second, we adopt a version of visible states semantics [45,25,38], treating all executions within a module as atomic. We only record runtime configurations which are *external* to module M, *i.e.* those where the executing object (*i.e.* the current receiver) comes from module $\texttt{M}'$. Execution has the form

$$\texttt{M} \,;\, \texttt{M}', \sigma \leadsto \sigma'$$

where we ignore all intermediate steps with receivers internal to M. In the next section we shall outline the underlying programming language, and define the judgment $\texttt{M} \,;\, \texttt{M}', \sigma \leadsto \sigma'$ and the set $\mathcal{A}rising(\texttt{M} \,;\, \texttt{M}')$.

### 4.2 An underlying programming language, $\mathcal{L}_{oo}$

The meaning of $\mathcal{C}hainmail$ assertions is parametric with an underlying object-oriented programming language, with modules as repositories of code, classes with fields, methods and ghostfields, objects described by classes, a way to link modules into larger ones, and a concept of program execution[6].

[6] We believe that $\mathcal{C}hainmail$ can be applied to any language with these features.

We have developed $\mathcal{L}_{oo}$, a minimal such object-oriented language, which we outline in this section. We describe the novel aspects of $\mathcal{L}_{oo}$, and summarise the more conventional parts, relegating full, and mostly unsurprising, definitions to Appendix A.

Modules are central to $\mathcal{L}_{oo}$, as they are to $\mathcal{C}hainmail$. As modules are repositories of code, we adopt the common formalisation of modules as maps from class identifiers to class definitions, c.f. Appendix, Def. 1. We use the terms module and component in an analogous manner to class and object respectively. $\mathcal{L}_{oo}$ is untyped for several reasons. Many popular programming languages are untyped. The external module might be untyped, and so it is more general to consider everything as untyped. Finally, a solution that works for an untyped language will also apply to a typed language, while the converse is not true.

Class definitions consist of field, method and ghost field declarations, c.f. Appendix, Def. 2. Method bodies are sequences of statements, which can be field reads or field assignments, object creation, method calls, and return statements. Fields are private in the sense of C++: they can only be read or written by methods of the current class. This is enforced by the operational semantics, *c.f.* Fig. 1. We discuss ghost fields in the next section.

Runtime configurations, $\sigma$, contain all the usual information about execution snapshots: the heap, and a stack of frames. Each frame consists of a continuation, `contn`, describing the remaining code to be executed by the frame, and a map from variables to values. Values are either addresses or sets of addresses; sets are needed to deal with assertions which quantify over sets of objects, such as assertions (1) and (2) from section 2. We define *one-module* execution through a judgment of the form $\mathtt{M}, \sigma \rightsquigarrow \sigma'$ in the Appendix, Fig. 1.

We define a module linking operator $\circ$ so that $\mathtt{M}\circ\mathtt{M}'$ is the union of the two modules, provided that their domains are disjoint, c.f. Appendix, Def. 8. As we said in section 4.1, we distinguish between the internal and external module. We consider execution from the view of the external module, and treat execution of methods from the internal module as atomic. For this, we define *two-module execution* based on one-module execution as follows:

**Definition 1.** *Given runtime configurations $\sigma$, $\sigma'$, and a module-pair $\mathtt{M} ⨾ \mathtt{M}'$ we define execution where $\mathtt{M}$ is the internal, and $\mathtt{M}'$ is the external module as below:*

- $\mathtt{M} ⨾ \mathtt{M}', \sigma \rightsquigarrow \sigma'$ *if there exist $n \geq 2$ and runtime configurations $\sigma_1$, ... $\sigma_n$, such that*
  - $\sigma = \sigma_1$, *and* $\sigma_n = \sigma'$.
  - $\mathtt{M}\circ\mathtt{M}', \sigma_i \rightsquigarrow \sigma'_{i+1}$, *for* $1 \leq i \leq n-1$
  - $\mathcal{C}lass(\mathtt{this})_\sigma \notin dom(\mathtt{M})$, *and* $\mathcal{C}lass(\mathtt{this})_{\sigma'} \notin dom(\mathtt{M})$,
  - $\mathcal{C}lass(\mathtt{this})_{\sigma_i} \in dom(\mathtt{M})$, *for* $2 \leq i \leq n-2$

In the definition above, $\mathcal{C}lass(\mathtt{x})_\sigma$ looks up the class of the object stored at `x`, c.f. Appendix, Def. 5. For example, for $\sigma_4$ as in Section 3 whose next statement to be executed is `a`$_2$`.deposit(a`$_3$`, 360)`, we would have a sequence of configurations $\sigma_{41}$, ... $\sigma_{4n}$, $\sigma_5$ so that the one-module execution gives $\mathtt{M}_{BA2}, \sigma_4 \rightsquigarrow \sigma_{41} \rightsquigarrow \sigma_{42} ... \rightsquigarrow$

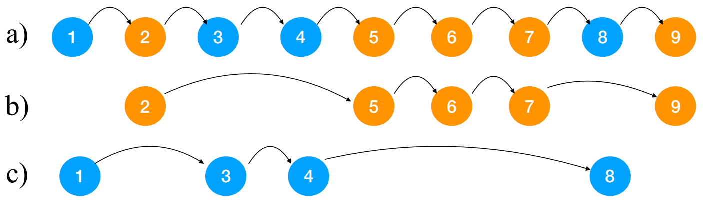

**Figure 3.** Two Module Execution (Def. 1). a) $\mathtt{M}_1 \circ \mathtt{M}_2$ b) $\mathtt{M}_1 \,⨟\, \mathtt{M}_2$ c) $\mathtt{M}_2 \,⨟\, \mathtt{M}_1$

$\sigma_{4n} \leadsto \sigma_5$. This would correspond to an atomic evaluation in the two-module execution: $\mathtt{M}_{BA2} \,⨟\, \mathtt{M}', \sigma_4 \leadsto \sigma_5$ (see Fig.3; where blue stands for $\sigma(this) \in M_1$,and orange for $\sigma(this) \in M_2$).

Two-module execution is related to visible states semantics [45] as they both filter configurations, with the difference that in visible states semantics execution is unfiltered and configurations are only filtered when it comes to the consideration of class invariants while two-module execution filters execution. The lemma below says that linking is associative and commutative, and preserves both one-module and two-module execution.

**Lemma 1 (Properties of linking).** *For any modules* $\mathtt{M}$, $\mathtt{M}'$, $\mathtt{M}''$, *and* $\mathtt{M}'''$ *and runtime configurations* $\sigma$, *and* $\sigma'$ *we have*:

– $(\mathtt{M}\circ\mathtt{M}')\circ\mathtt{M}'' = \mathtt{M}\circ(\mathtt{M}'\circ\mathtt{M}'')$ *and* $\mathtt{M}\circ\mathtt{M}' = \mathtt{M}'\circ\mathtt{M}$.
– $\mathtt{M}, \sigma \leadsto \sigma'$, *and* $\mathtt{M}\circ\mathtt{M}'$ *is defined,* *implies* $\mathtt{M}\circ\mathtt{M}', \sigma \leadsto \sigma'$.
– $\mathtt{M} \,⨟\, \mathtt{M}', \sigma \leadsto \sigma'$ *implies* $(\mathtt{M}\circ\mathtt{M}'') \,⨟\, (\mathtt{M}'\circ\mathtt{M}'''), \sigma \leadsto \sigma'$.

We can now answer the question as to which runtime configurations are pertinent when judging a module's adherence to an assertion. *Initial configurations* are those whose heap have only one object, of class `Object`, and whose stack have one frame, with arbitrary continuation. *Arising* configurations are those that can be reached by two-module execution, starting from any initial configuration.

**Definition 2 (Initial and Arising Configurations).** *are defined as follows:*

– $\mathcal{I}nitial\langle(\psi, \chi)\rangle$, *if* $\psi$ *consists of a single frame* $\phi$ *with* $dom(\phi) = \{\mathtt{this}\}$, *and there exists some address* $\alpha$, *such that* $\lfloor\mathtt{this}\rfloor_\phi = \alpha$, *and* $dom(\chi) = \alpha$, *and* $\chi(\alpha) = (\mathtt{Object}, \emptyset)$.
– $\mathcal{A}rising(\mathtt{M} \,⨟\, \mathtt{M}') = \{\, \sigma \mid \exists \sigma_0.\, [\, \mathcal{I}nitial\langle\sigma_0\rangle \;\wedge\; \mathtt{M} \,⨟\, \mathtt{M}', \sigma_0 \leadsto^* \sigma \,]\, \}$

## 5 Assertions

*Chainmail* assertions (details in appendix B.3) consist of (pure) expressions `e`, comparisons between expressions, classical assertions about the contents of heap and stack, the usual logical connectives, as well as our holistic concepts. In this section we focus on the novel, holistic, features of *Chainmail* (permission, control, time, space, and viewpoint), as well as our wish to support some form of recursion while keeping the logic of assertions classical.

### 5.1 Satisfaction of Assertions - Access, Control, Space, Viewpoint

*Permission* expresses that an object has the potential to call methods on another object, and to do so directly, without help from any intermediary object. This is the case when the two objects are aliases, or the first object has a field pointing to the second object, or the first object is the receiver of the currently executing method and the second object is one of the arguments or a local variable. Interpretations of variables and paths, $\lfloor ... \rfloor_\sigma$, are defined in the usual way (appendix Def. 5).

**Definition 3 (Permission).** *For any modules* M, M′*, variables* x *and* y*, we define*

- $\texttt{M} \,⨟\, \texttt{M}', \sigma \models \langle\, \texttt{x}\ \textsf{access}\ \texttt{y} \,\rangle$ *if* $\lfloor \texttt{x} \rfloor_\sigma$ *and* $\lfloor \texttt{y} \rfloor_\sigma$ *are defined, and*
  - $\lfloor \texttt{x} \rfloor_\sigma = \lfloor \texttt{y} \rfloor_\sigma$, *or*
  - $\lfloor \texttt{x.f} \rfloor_\sigma = \lfloor \texttt{y} \rfloor_\sigma$, *for some field* f, *or*
  - $\lfloor \texttt{x} \rfloor_\sigma = \lfloor \texttt{this} \rfloor_\sigma$ *and* $\lfloor \texttt{y} \rfloor_\sigma = \lfloor \texttt{z} \rfloor_\sigma$, *for some variable* z *and* z *appears in* $\sigma.\texttt{contn}$.

In the last disjunct, where z is a parameter or local variable, we ask that z appears in the code being executed ($\sigma.\texttt{contn}$). This requirement ensures that variables which were introduced into the variable map in order to give meaning to existentially quantified assertions, are not considered.

*Control* expresses which object is the process of making a function call on another object and with what arguments. The relevant information is stored in the continuation (cont) on the top frame.

**Definition 4 (Control).** *For any modules* M, M′*, variables* x , y, $\texttt{z}_1, ...\texttt{z}_n$*, we define*:

- $\texttt{M} \,⨟\, \texttt{M}', \sigma \models \langle\, \texttt{x}\ \textsf{calls}\ \texttt{y.m}(\ \texttt{z}_1, ...\texttt{z}_n)\ \rangle$ *if* $\lfloor \texttt{x} \rfloor_\sigma$, $\lfloor \texttt{y} \rfloor_\sigma$, $\lfloor \texttt{z}_1 \rfloor_\sigma$, ... $\lfloor \texttt{z}_n \rfloor_\sigma$ *are defined, and*
  - $\lfloor \texttt{this} \rfloor_\sigma = \lfloor \texttt{x} \rfloor_\sigma$, *and*
  - $\sigma.\texttt{contn} = \texttt{u.m}(\texttt{v}_1, ..\texttt{v}_n); \_$, *for some* $\texttt{u}, \texttt{v}_1, ...\ \texttt{v}_n$, *and*
  - $\lfloor \texttt{y} \rfloor_\sigma = \lfloor \texttt{u} \rfloor_\sigma$, *and* $\lfloor \texttt{z}_i \rfloor_\sigma = \lfloor \texttt{v}_i \rfloor_\sigma$, *for all i.*

Thus, $\langle\, \texttt{x}\ \textsf{calls}\ \texttt{y.m}(\ \texttt{z}_1, ...\texttt{z}_n)\ \rangle$ expresses the call $\texttt{y.m}(\texttt{z}_1, ...\texttt{z}_n)$ will be executed next, and that the caller is x.

*Viewpoint* is about whether an object is viewed as belonging to the internal mode; this is determined by the class of the object.

**Definition 5 (Viewpoint).** *For any modules* M, M′*, and variable*x*, we define*

- $\texttt{M} \,⨟\, \texttt{M}', \sigma \models \textsf{external}\langle\, \texttt{x} \,\rangle$ *if* $\lfloor \texttt{x} \rfloor_\sigma$ *is defined and* $Class(\lfloor \texttt{x} \rfloor_\sigma)_\sigma \notin dom(\texttt{M})$
- $\texttt{M} \,⨟\, \texttt{M}', \sigma \models \textsf{internal}\langle\, \texttt{x} \,\rangle$ *if* $\lfloor \texttt{x} \rfloor_\sigma$ *is defined and* $Class(\lfloor \texttt{x} \rfloor_\sigma)_\sigma \in dom(\texttt{M})$

*Space* is about asserting that some property $A$ holds in a configuration whose objects are restricted to those from a given set S. This way we can express that the objects from the set S have authority over the assertion $A$. In order to define validity of $\langle\, A\ \textsf{in}\ \texttt{S} \,\rangle$ in a configuration $\sigma$, we first define a restriction operation, $\sigma{\downarrow}_{\texttt{S}}$ which restricts the objects from $\sigma$ to only those from S.

**Definition 6 (Restriction of Runtime Configurations).** *The restriction operator* $\downarrow$ *applied to a runtime configuration* $\sigma$ *and a variable* $\mathtt{S}$ *is defined as follows:*

– $\sigma\downarrow_{\mathtt{S}} \triangleq (\psi, \chi')$, *if* $\sigma=(\psi,\chi)$, $dom(\chi') = \lfloor \mathtt{S} \rfloor_\sigma$, *and* $\forall \alpha \in dom(\chi').\chi(\alpha) = \chi'(\alpha)$.

For example, if we take $\sigma_2$ from Fig. 2 in Section 2, and restrict it with some set $\mathtt{S}_4$ such that $\lfloor \mathtt{S}_4 \rfloor_{\sigma_2} = \{91, 1, 2, 3, 4, 11\}$, then the restriction $\sigma_2\downarrow_{\mathtt{S}_4}$ will look as on the right.

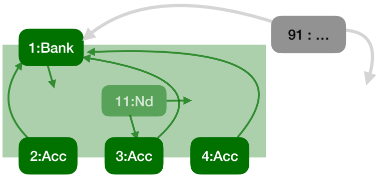

Note in the diagram above the dangling pointers at objects 1, 11, and 91 - reminiscent of the separation of heaps into disjoint subheaps, as provided by the $*$ operator in separation logic [53]. The difference is that in separation logic, the separation is provided through the assertions, where $A * A'$ holds in any heap which can be split into disjoint $\chi$ and $\chi'$ where $\chi$ satisfies $A$ and $\chi'$ satisfies $A'$. That is, in $A * A'$ the split of the heap is determined by the assertions $A$ and $A'$ and there is an implicit requirement of disjointness, while in $\sigma\downarrow_{\mathtt{S}}$ the split is determined by $\mathtt{S}$, and no disjointness is required.

We now define the semantics of $\langle\, A \;\mathsf{in}\; \mathtt{S} \,\rangle$.

**Definition 7 (Space).** *For any modules* $\mathtt{M}$, $\mathtt{M}'$, *assertions* $A$ *and variable* $\mathtt{S}$, *we define*:

– $\mathtt{M} \,⨟\, \mathtt{M}', \sigma \models \langle\, A \;\mathsf{in}\; \mathtt{S} \,\rangle$ *if* $\mathtt{M} \,⨟\, \mathtt{M}', \sigma\downarrow_{\mathtt{S}} \models A$.

The set $\mathtt{S}$ in the assertion $\langle\, A \;\mathsf{in}\; \mathtt{S} \,\rangle$ is related to framing from implicit dynamic frames [57]: in an implicit dynamic frames assertion **acc** $\mathtt{x.f} * A$, the frame $\mathtt{x.f}$ prescribes which locations may be used to determine validity of $A$. The difference is that frames are sets of locations (pairs of address and field), while our $\mathtt{S}$-es are sets of addresses. More importantly, implicit dynamic frames assertions whose frames are not large enough are badly formed, while in our work, such assertions are allowed and may hold or not, *e.g.* $\mathtt{M}_{BA2} \,⨟\, \mathtt{M}', \sigma \models \neg \langle\, (\exists n.\mathtt{a_2.balance} = n) \;\mathsf{in}\; \mathtt{S}_4 \,\rangle$.

## 5.2 Satisfaction of Assertions - Time

To deal with time, we are faced with four challenges: a) validity of assertions in the future or the past needs to be judged in the future configuration, but using the bindings from the current one, b) the current configuration needs to store the code being executed, so as to be able to calculate future configurations, c) when considering the future, we do not want to observe configurations which go beyond the frame currently at the top of the stack, d) there is no "undo" operator to deterministically enumerate all the previous configurations.

Consider challenge a) in some more detail: the assertion $\mathsf{will}\langle\, \mathtt{x.f} = 3 \,\rangle$ is satisfied in the *current* configuration $\sigma_1$, if in some *future* configuration $\sigma_2$, the field $\mathtt{f}$ of the object that is pointed at by $\mathtt{x}$ in the *current* configuration ($\sigma_1$) has the value $3$, that is, if $\lfloor \lfloor \mathtt{x} \rfloor_{\sigma_1}.f \rfloor_{\sigma_2} = 3$, even if in that future configuration $\mathtt{x}$ denotes a different object (i.e. if $\lfloor \mathtt{x} \rfloor_{\sigma_1} \neq \lfloor \mathtt{x} \rfloor_{\sigma_2}$). To address this, we define an auxiliary concept: the operator $\lhd$, where

$\sigma_1 \triangleleft \sigma_2$ adapts the second configuration to the top frame's view of the former: it returns a new configuration whose stack comes from $\sigma_2$ but is augmented with the view from the top frame from $\sigma_1$ and where the continuation has been consistently renamed. This allows us to interpret expressions in $\sigma_2$ but with the variables bound according to $\sigma_1$; *e.g.* we can obtain that value of x in configuration $\sigma_2$ even if x was out of scope in $\sigma_2$.

**Definition 8 (Adaptation).** *For runtime configurations $\sigma_1$, $\sigma_2$.:*

- $\sigma_1 \triangleleft \sigma_2 \triangleq (\phi_3 \cdot \psi_2, \chi_2)$ *if*
  - $\phi_3 = (\, \texttt{contn}_2[\texttt{zs}_2/\texttt{zs}'], \beta_2[\texttt{zs}' \mapsto \beta_2(\texttt{zs}_2)][\texttt{zs}_1 \mapsto \beta_1(\texttt{zs}_1)]\,)$, *where*
  - $\sigma_1 = (\phi_1 \cdot \_, \_)$, $\sigma_2 = (\phi_2 \cdot \psi_2, \chi_2)$, $\phi_1 = (\_, \beta_1)$, $\phi_2 = (\texttt{contn}_2, \beta_2)$, *and*
  - $\texttt{zs}_1 = dom(\beta_1)$, $\texttt{zs}_2 = dom(\beta_2)$, *and*
  - $\texttt{zs}'$ *is a set of variables with the same cardinality as* $\texttt{zs}_2$, *and all variables in* $\texttt{zs}'$ *are fresh in* $\beta_1$ *and in* $\beta_2$.

That is, in the new frame $\phi_2$ from above, we keep the same continuation as from $\sigma_2$ but rename all variables with fresh names $\texttt{zs}'$, and combine the variable map $\beta_1$ from $\sigma_1$ with the variable map $\beta_2$ from $\sigma_2$ while avoiding names clashes through the renaming $[\texttt{zs}' \mapsto \beta_2(\texttt{zs}_2)]$. The consistent renaming of the continuation allows the correct modelling of execution, as needed for the semantics of nested time assertions, as *e.g.* in $\mathsf{will}\langle\, \texttt{x.f} = 3 \wedge \mathsf{will}\langle\, \texttt{x.f} = 5 \,\rangle\,\rangle$.

Having addressed challenge a) we turn our attention to the remaining challenges: We address challenge b) by storing the remaining code to be executed in cntn in each frame. We address challenge c) by only taking the top of the frame when considering future executions. Finally, we address challenge d) by considering only configurations which arise from initial configurations, and which lead to the current configuration.

**Definition 9 (Time Assertions).** *For any modules* M, M′*, and assertion A we define*

- $\texttt{M} \fatsemi \texttt{M}', \sigma \models \mathsf{next}\langle\, A \,\rangle$ *if* $\exists \sigma'.[\ \texttt{M} \fatsemi \texttt{M}', \phi \rightsquigarrow \sigma' \ \wedge \texttt{M} \fatsemi \texttt{M}', \sigma \triangleleft \sigma' \models A\ ]$, *and where $\phi$ is so that* $\sigma = (\phi \cdot \_, \_)$.
- $\texttt{M} \fatsemi \texttt{M}', \sigma \models \mathsf{will}\langle\, A \,\rangle$ *if* $\exists \sigma'.[\ \texttt{M} \fatsemi \texttt{M}', \phi \rightsquigarrow^* \sigma' \ \wedge \texttt{M} \fatsemi \texttt{M}', \sigma \triangleleft \sigma' \models A\ ]$, *and where $\phi$ is so that* $\sigma = (\phi \cdot \_, \_)$.
- $\texttt{M} \fatsemi \texttt{M}', \sigma \models \mathsf{prev}\langle\, A \,\rangle$ *if* $\forall \sigma_1, \sigma_2.[\ \mathit{Initial}\langle \sigma_1 \rangle \ \wedge\ \texttt{M} \fatsemi \texttt{M}', \sigma_1 \rightsquigarrow^* \sigma_2 \ \wedge \texttt{M} \fatsemi \texttt{M}', \sigma_2 \rightsquigarrow \sigma \ \longrightarrow\ \texttt{M} \fatsemi \texttt{M}', \sigma \triangleleft \sigma_2 \models A\ ]$
- $\texttt{M} \fatsemi \texttt{M}', \sigma \models \mathsf{was}\langle\, A \,\rangle$ *if* $\forall \sigma_1.[\ \mathit{Initial}\langle \sigma_1 \rangle \ \wedge\ \texttt{M} \fatsemi \texttt{M}', \sigma_1 \rightsquigarrow^* \sigma \longrightarrow (\ \exists \sigma_2. \texttt{M} \fatsemi \texttt{M}', \sigma_1 \rightsquigarrow^* \sigma_2 \ \wedge\ \texttt{M} \fatsemi \texttt{M}', \sigma_2 \rightsquigarrow^* \sigma \ \wedge\ \texttt{M} \fatsemi \texttt{M}', \sigma \triangleleft \sigma_2 \models A\ )]$

In general, $\mathsf{will}\langle\, \langle\, A\ \mathsf{in}\ \texttt{S} \,\rangle\,\rangle$ is different from $\langle\, \mathsf{will}\langle\, A \,\rangle\ \mathsf{in}\ \texttt{S} \,\rangle$. In the former assertion, S must contain the objects involved in reaching the future configuration as well as the objects needed to then establish validity of $A$ in that future configuration. In the latter assertion, S need only contain the objects needed to establish $A$ in that future configuration. For example, revisit Fig. 2, and take $\texttt{S}_1$ to consist of objects 1, 2, 4, 93, and 94, and $\texttt{S}_2$ to consist of objects 1, 2, 4. Assume that $\sigma_5$ is like $\sigma_1$, that the next call in $\sigma_5$ is a method on $\texttt{u}_{94}$, whose body obtains the address of $\texttt{a}_4$ (by making a call on 93 to which it has access), and the address of $\texttt{a}_2$ (to which it has access), and then makes the call $\texttt{a}_2.\texttt{deposit}(\texttt{a}_4, 360)$. Assume also that $\texttt{a}_4$'s balance is 380. Then

$$\texttt{M}_{BA1} \fatsemi ..., \sigma_5 \ \models\ \langle\, \mathsf{will}\langle\, \mathsf{changes}\langle\, \texttt{a}_2.\texttt{balance} \,\rangle\,\rangle\ \mathsf{in}\ \texttt{S}_1 \,\rangle$$
$$\texttt{M}_{BA1} \fatsemi ..., \sigma_5 \ \not\models\ \langle\, \mathsf{will}\langle\, \mathsf{changes}\langle\, \texttt{a}_2.\texttt{balance} \,\rangle\,\rangle\ \mathsf{in}\ \texttt{S}_2 \,\rangle$$
$$\texttt{M}_{BA1} \fatsemi ..., \sigma_5 \ \models\ \mathsf{will}\langle\, \langle\, \mathsf{changes}\langle\, \texttt{a}_2.\texttt{balance} \,\rangle\ \mathsf{in}\ \texttt{S}_2 \,\rangle\,\rangle$$

### 5.3 Properties of Assertions

We define equivalence of assertions in the usual way: assertions $A$ and $A'$are equivalent if they are satisfied in the context of the same configurations and module pairs – *i.e.*

$$A \equiv A' \quad \text{if} \quad \forall \sigma. \forall \mathtt{M}, \mathtt{M}'. [\ \mathtt{M} \mathbin{⨾} \mathtt{M}', \sigma \models A \ \text{ if and only if } \ \mathtt{M} \mathbin{⨾} \mathtt{M}', \sigma \models A'\ ].$$

We can then prove that the usual equivalences hold, *e.g.* $A \vee A' \equiv A' \vee A$, and $\neg(\exists \mathtt{x}.A) \equiv \forall \mathtt{x}.(\neg A)$. Our assertions are classical, *e.g.* $A \wedge \neg A \equiv \mathtt{false}$, and $\mathtt{M} \mathbin{⨾} \mathtt{M}', \sigma \models A$ and $\mathtt{M} \mathbin{⨾} \mathtt{M}', \sigma \models A \rightarrow A'$ implies $\mathtt{M} \mathbin{⨾} \mathtt{M}', \sigma \models A'$. This desirable property comes at the loss of some expected equivalences, *e.g.*, in general, $\mathtt{e} = \mathtt{false}$ and $\neg \mathtt{e}$ are not equivalent. More in Appendix B.

### 5.4 Modules satisfying assertions

Finally, we define satisfaction of assertions by modules: a module $\mathtt{M}$ satisfies an assertion $A$ if for all other potential modules $\mathtt{M}'$, in all configurations arising from executions of $\mathtt{M} \mathbin{⨾} \mathtt{M}'$, the assertion $A$ holds.

**Definition 10.** *For any module* $\mathtt{M}$*, and assertion* $A$*, we define:*

– $\mathtt{M} \models A \quad \textit{if} \quad \forall \mathtt{M}'. \forall \sigma \in \mathcal{A}rising(\mathtt{M} \mathbin{⨾} \mathtt{M}'). \ \mathtt{M} \mathbin{⨾} \mathtt{M}', \sigma \models A$

## 6 Examplar Driven Design

*Examplars* The design of *Chainmail* was guided by the study of a sequence of exemplars taken from the object-capability literature and the smart contracts world:

1. **Bank** [49] - Bank and Account as in Section 2 with two different implementations.
2. **ERC20** [61] - Ethereum-based token contract.
3. **DAO** [12,15] - Ethereum contract for Decentralised Autonomous Organisation.
4. **DOM** [20,59] - Restricting access to browser Domain Object Model

We present these exemplars as appendices [1]. Our design was also driven by work on other examples such as the membrane [17], the Mint/Purse [40], and Escrow [18,24].

*Model* We have constructed a Coq model[7] [23] of the core of the Chainmail specification language, along with the underlying $\mathcal{L}_{oo}$ language. Our formalism is organised as follows:

1. The $\mathcal{L}_{oo}$ Language: a class based, object oriented language with mutable references.
2. Chainmail: The full assertion syntax and semantics defined in Definitions 1, 2, 3, 4, 5, 6, 7, 8, 9 and 10.
3. $\mathcal{L}_{oo}$ Properties: Secondary properties of the loo language that aid in reasoning about its semantics.
4. Chainmail Properties: The core properties defined on the semantics of Chainmail.

[7] A current model can be found at: https://github.com/sophiaIC/HolisticSpecifications

In the associated appendix (see Appendix G) we list and present the properties of Chainmail we have formalised in Coq. We have proven that Chainmail obeys much of the properties of classical logic. While we formalise most of the underlying semantics, we make several assumptions in our Coq formalism: (i) the law of the excluded middle, a property that is well known to be unprovable in constructive logics, and (ii) the equality of variable maps and heaps down to renaming. Coq formalisms often require fairly verbose definitions and proofs of properties involving variable substitution and renaming, and assuming equality down to renaming saves much effort.

More details of the formal foundations of $\mathcal{C}hainmail$, and the model, are also in appendices [1].

## 7 Related Work

*Behavioural Specification Languages* Hatcliff et al. [26] provide an excellent survey of contemporary specification approaches. With a lineage back to Hoare logic [28], Meyer's Design by Contract [38] was the first popular attempt to bring verification techniques to object-oriented programs as a "whole cloth" language design in Eiffel. Several more recent specification languages are now making their way into practical and educational use, including JML [31], Spec♯ [4], Dafny [32] and Whiley [51]. Our approach builds upon these fundamentals, particularly Leino & Shulte's formulation of two-state invariants [33], and Summers and Drossopoulou's Considerate Reasoning [58]. In general, these approaches assume a closed system, where modules can be trusted to coöperate. In this paper we aim to work in an open system where modules' invariants must be protected irrespective of the behaviour of the rest of the system.

*Defensive Consistency* In an open world, we cannot rely on the kindness of strangers: rather we have to ensure our code is correct regardless of whether it interacts with friends or foes. Attackers *"only have to be lucky once"* while secure systems *"have to be lucky always"* [5]. Miller [39,40] defines the necessary approach as **defensive consistency**: *"An object is defensively consistent when it can defend its own invariants and provide correct service to its well behaved clients, despite arbitrary or malicious misbehaviour by its other clients."* Defensively consistent modules are particularly hard to design, to write, to understand, and to verify: but they make it much easier to make guarantees about systems composed of multiple components [46].

*Object Capabilities and Sandboxes.* *Capabilities* as a means to support the development of concurrent and distributed system were developed in the 60's by Dennis and Van Horn [19], and were adapted to the programming languages setting in the 70's [44]. *Object capabilities* were first introduced [40] in the early 2000s, and many recent studies manage to verify safety or correctness of object capability programs. Google's Caja [42] applies sandboxes, proxies, and wrappers to limit components' access to *ambient* authority. Sandboxing has been validated formally: Maffeis et al. [35] develop a model of JavaScript, demonstrate that it obeys two principles of object capability systems and show how untrusted applications can be prevented from interfering with the rest of the system. Recent programming languages [27,10,54] including Newspeak [9], Dart [8], Grace [7,30] and Wyvern [36] have adopted the object capability model.

*Verification of Object Capability Programs* Murray made the first attempt to formalise defensive consistency and correctness [46]. Murray's model was rooted in counterfactual causation [34]: an object is defensively consistent when the addition of untrustworthy clients cannot cause well-behaved clients to be given incorrect service. Murray formalised defensive consistency very abstractly, over models of (concurrent) object-capability systems in the process algebra CSP [29], without a specification language for describing effects, such as what it means for an object to provide incorrect service. Both Miller and Murray's definitions are intensional, describing what it means for an object to be defensively consistent.

Drossopoulou and Noble [21,48] have analysed Miller's Mint and Purse example [40] and discussed the six capability policies as proposed in [40]. In [22], they sketched a specification language, used it to specify the six policies from [40], showed that several possible interpretations were possible, and uncovered the need for another four further policies. They also sketched how a trust-sensitive example (the escrow exchange) could be verified in an open world [24]. Their work does not support the concepts of control, time, or space, as in $\mathcal{Chainmail}$, but it offers a primitive expressing trust.

Devriese et al. [20] have deployed powerful theoretical techniques to address similar problems: They show how step-indexing, Kripke worlds, and representing objects as state machines with public and private transitions can be used to reason about object capabilities. Devriese have demonstrated solutions to a range of exemplar problems, including the DOM wrapper (replicated in our section F) and a mashup application. Their distinction between public and private transitions is similar to the distinction between internal and external objects.

More recently, Swasey et al. [59] designed OCPL, a logic for object capability patterns, that supports specifications and proofs for object-oriented systems in an open world. They draw on verification techniques for security and information flow: separating internal implementations ("high values" which must not be exposed to attacking code) from interface objects ("low values" which may be exposed). OCPL supports defensive consistency (they use the term "robust safety" from the security community [6]) via a proof system that ensures low values can never leak high values to external attackers. This means that low values *can* be exposed to external code, and the behaviour of the system is described by considering attacks only on low values. They use that logic to prove a number of object-capability patterns, including sealer/unsealer pairs, the caretaker, and a general membrane.

Schaefer et al. [55] have recently added support for information-flow security using refinement to ensure correctness (in this case confidentiality) by construction. By enforcing encapsulation, all these approaches share similarity with techniques such as ownership types [14,50], which also protect internal implementation objects from accesses that cross encapsulation boundaries. Banerjee and Naumann demonstrated that by ensuring confinement, ownership systems can enforce representation independence (a property close to "robust safety") some time ago [3].

$\mathcal{Chainmail}$ differs from Swasey, Schaefer's, and Devriese's work in a number of ways: They are primarily concerned with mechanisms that ensure encapsulation (aka confinement) while we abstract away from any mechanism via the $\mathsf{external}\langle\ \rangle$ predicate. They use powerful mathematical techniques which the users need to understand in order to write their specifications, while *Chainmail* users only need to understand first order logic and the holistic operators presented in this paper. Finally, none of these systems offer the kinds of holistic assertions addressing control flow, change, or temporal operations that are at the core of *Chainmail*'s approach.

Scilla [56] is a minimalistic typed functional language for writing smart contracts that compiles to the Ethereum bytecode. Scilla's semantic model is restricted, assuming actor based communication and restricting recursion, thus facilitating static analysis of Scilla contracts and ensuring termination. Scilla is able to demonstrate that a number of popular Ethereum contracts avoid type errors, out-of-gas resource failures, and preservation of virtual currency. Scilla's semantics are defined formally, but have not yet been represented in a mechanised model.

Finally, the recent VerX tool is able to verify a range of specifications for solidity contracts automatically [52]. Similar to *Chainmail*, VerX has a specification language based on temporal logic. VerX offers three temporal operators (always, once, prev) but only within a past modality, while *Chainmail* has two temporal operators, both existential, but with both past and future modalities. VerX specifications can also include predicates that model the current invocation on a contract (similar to *Chainmail*'s "calls"), can access variables, and compute sums (only) over collections. *Chainmail* is strictly more expressive as a specification language, including quantification over objects and sets (so can compute arbitrary reductions on collections) and of course specifications for permission ("access"), space ("in") and viewpoint ("external") which have no analogues in VerX. Unlike *Chainmail*, VerX includes a practical tool that has been used to verify a hundred properties across case studies of twelve Solidity contracts.

## 8 Conclusions

In this paper we have motivated the need for holistic specifications, presented the specification language *Chainmail* for writing such specifications, and outlined the formal foundations of the language. To focus on the key attributes of a holistic specification language, we have kept *Chainmail* simple, only requiring an understanding of first order logic. We believe that the holistic features (permission, control, time, space and viewpoint) are intuitive concepts when reasoning informally, and were pleased to have been able to provide their formal semantics in what we argue is a simple manner.

## 9 Acknowledgments

This work is based on a long-standing collaboration with Mark S. Miller and Toby Murray. We have received invaluable feedback from Alex Summers, Bart Jacobs, Chris Hawblitzel, Michael Jackson, Lucius G. Meredith, Mike Stay, Shuh Peng Loh, Emil Klasan, members of WG 2.3, and the FASE 2020 reviewers. The work has been supported by the Royal Society of New Zealand (Te Apārangi) Marsden Fund (Te Pūtea Rangahau a Marsden) grants VUW-1318 and VUW-1815, and research gifts from Agoric, the Ethereum Foundation, and Facebook.
